# Volunteer Surgeons

**Published:** 1862-09

**Authors:** 


Volunteer Surgeons.—The Mayor of Buffalo and District War Committee have asked for ten, or more, Volunteer Surgeons, to leave immediately for the seat of war. The required number have at once volunteered and are ready for immediate service.

